# Supplementary material for: Are carbon emissions trading and green financial instruments synergistic? -Comprehensive quantitative research based on content analysis
Source: PLoS One. 2024 Mar 7;19(3):e0298601. doi: 10.1371/journal.pone.0298601 (PMC10919601; doi:10.1371/journal.pone.0298601)
Supplement: S1 Data — (ZIP) [file pone.0298601.s001.zip › Data/地级市Z_F工作报告.zip!/║╙▒▒/╠╞╔╜╩╨/└┤╫╘╣1⁄2╓┌║┼í╛┬φ┐╦╩2╛▌═°í┐_2017.docx]

十四届政府及2016年工作回顾

2016年是唐山人民备受鼓舞、倍感自豪，具有里程碑意义的一年。一年来，我们把稳增长作为首要任务，以增量培育和有效投资推动经济平稳较快发展，展现了负重奋进、攻坚克难的唐山作为。一年来，我们把加快新旧动能转换作为主攻方向，以去产能打开供给侧结构性改革突破口，彰显了勇闯新路、主动担当的唐山责任。一年来，我们把系列重大活动作为起飞点，以“三城联动”加速资源型城市转型，塑树了开放包容、活力迸发的唐山形象。一年来，我们把打好“沿海”“京津”两张牌作为战略重点，以释放沿海潜能加速临港产业聚集，开掘了依托大港、走向世界的唐山优势。一年来，我们把狠抓牵动全局的着力点作为关键举措，以重点领域创新突破激发活力动力，形成了谋实抓深、精准发力的唐山共识。一年来，我们把保障和改善民生作为落脚点，以惠民实事增强人民群众的获得感，提升了以人为本、共建共享的唐山温度。

市十四届人大一次会议以来，全市上下深入学习贯彻习近平总书记系列重要讲话精神，在省委、省政府和市委的坚强领导下，面对复杂严峻的国内外经济形势，主动适应和把握新常态，积极应对经济下行巨大压力，全力以赴稳增长、调结构、促改革、惠民生、防风险，开创了唐山经济社会发展新局面，实现了“十二五”圆满收官和“十三五”良好开局。

一是综合实力持续增强。全市地区生产总值达到6306.2亿元，年均增长6.4%，经济总量居全省首位。固定资产投资累计完成1.7万亿元，年均增长14.8%。一般公共预算收入355.1亿元，是2012年的1.2倍。

二是转型升级步伐加快。三次产业比重由9.0∶59.3∶31.7优化为9.5:54.1:36.4。高新技术产业和装备制造业增加值增长高于规模以上工业增速，占比分别提高3.9个和7.1个百分点。服务业对经济增长贡献率达到53.5%，比2012年提高24.2个百分点。农业产业化经营率提高到68.4%。

三是沿海经济活力显现。唐山港货物吞吐量5.2亿吨，其中集装箱193万标箱、是2012年的4.3倍。沿海板块产业聚集提速突破，一般公共预算收入占全市比重比2012年提高7.7个百分点。

四是城乡面貌显著改善。城市承载力辐射力不断增强，美丽乡村建设扎实推进，常住人口城镇化率提高到60.41%。张唐铁路、205国道改建、唐曹公路等竣工通车，唐山客车线正式运营，铁路、高速路网密度居全省前列。森林覆盖率达到36.6%，率先实现省级园林城市全覆盖。

五是改革开放成效明显。市本级行政权力由4227项消减到1776项、精简58%，市县乡三级网上审批实现全覆盖。实施世园会建设等PPP项目19个，撬动民间资本212亿元。2016年实际利用外资、对外投资分别达到14.8亿美元、3.9亿美元，是2012年的1.2倍和2.2倍。

六是民生福祉日益提升。城镇和农村居民人均可支配收入达到33725元和15023元，分别居全省第2位和第1位。城镇累计新增就业39万人，城乡居民基本医疗保险制度完成整合，低保水平提前达到全面小康标准。空气达标天数增加94天。教育、卫生、文化、体育等社会事业蓬勃发展，精神文明和民主法治建设全面加强，蝉联全国文明城市、全国双拥模范城荣誉称号。安全生产、食品药品监管、社会治理、民族宗教、广播电视、新闻出版、外事侨务、妇女儿童、人民防空、防震减灾、老区建设、气象地理、史志档案、老龄、残疾人等事业取得新成绩。

今后五年的基本思路

今后五年政府工作的基本思路是：全面贯彻党的十八大和十八届三中、四中、五中、六中全会精神，深入学习贯彻习近平总书记系列重要讲话精神和治国理政新理念新思想新战略以及视察唐山重要指示要求，牢固树立和贯彻落实五大新发展理念，加快实现“三个努力建成”，在转变发展方式、调整经济结构、推进供给侧结构性改革等方面走在前列，打好“沿海”“京津”两张牌，推动要素投入增长型向创新驱动转变、资源依赖型发展向沿海开放拉动转变，做大做强城市经济、沿海增长极、县域经济“三大板块”，去钢铁产能，增新兴产业、增临港产业、增城市经济、增传统产业活力、增园区经济、增现代农业，加快建设国际化沿海强市。

今后五年的奋斗目标

奋力争取“三个走在前列”。到2021年，战略性新兴产业、服务业增加值年均增长14%和7%以上，三次产业结构更趋合理;全社会研发投入占GDP比重提高到2.5%以上，科技型中小企业达到8000家，高新技术企业突破420家，在同类城市中率先进入国家创新型城市行列;工业技改投资年均增长12%以上，钢铁高附加值产品比重达到30%以上，支柱产业80%的核心装备达到国际先进水平。

加快实现“三个努力建成”。到2021年，地区生产总值突破9000亿元，人均达到1.6万美元，一般公共预算收入达到531亿元;唐山港货物吞吐量达到6亿吨、集装箱突破500万标箱，开通国际国内航线200条以上，成为以大宗物流交易为主的综合性枢纽大港;综合保税区、中日韩循环经济示范基地等平台取得重大突破，东北亚经济合作窗口城市地位进一步凸显;高新技术产业、装备制造业增加值占规模以上工业比重分别达到27%和30%，高铁装备、新能源电池、特种机器人、石墨烯等产业成为京津冀乃至全国最大产业基地;中心城市建成区面积达到360平方公里、人口380万，常住人口城镇化率达到68.8%，成为京津冀区域中心城市和首都经济圈重要支点。

建设国际化沿海强市。到2021年，全市实际利用外资年均增长5%以上，引进省外资金年均增长10%以上;沿海地区生产总值、一般公共预算收入比2015年翻一番、年均增长15%以上，临港产业实现五年倍增;PM2.5年均浓度降至55微克/立方米，空气质量达标率达到65%以上，森林覆盖率达到40%;旅游、会展等产业快速发展，唐山成为国际会议目的地城市、区域性国际会展中心和中国北方重要的旅游休闲目的地;城乡居民人均可支配收入快于经济增长，基本公共服务水平和均等化走在全省前列，社会保障全民覆盖，社会文明程度明显提升，全面建成小康社会如期实现。

2017年工作安排

全市经济社会发展的主要目标是：地区生产总值增长6%以上，规模以上工业增加值增长5%以上，服务业增加值增长7.5%以上，一般公共预算收入增长7%，固定资产投资增长10%以上，社会消费品零售总额增长10%，出口总值增长5%，实际利用外资增长5%。每万元生产总值能耗和二氧化碳排放量、化学需氧量等主要污染物排放量均完成省达任务。常住人口城镇化率比上年提高1.95个百分点，城镇和农村居民人均可支配收入均增长7%，城镇登记失业率控制在4.5%以内，居民消费价格涨幅控制在3%以内。

围绕把市第十次党代会部署落实到年度，按照上述目标要求，着力抓好以下11个方面工作：

1、强化稳增长支撑，巩固扩大经济持续向好势头

一是压茬推进项目开工投产达效。今年市级重点实施亿元以上战略支撑项目300个，完成投资1300亿元。确保100个项目开工建设，100个项目投产达效。二是创新推动开发区提速突破。年内省级以上开发区直接利用外资和引进省外资金分别达到10亿美元和600亿元以上，主营业务收入、规模以上工业增加值、税收等主要指标明显高于全市平均水平。三是以基础设施建设拉动有效投资。全市实施500万元以上基础设施项目400个，完成投资600亿元以上。

2、推进工业经济转型提质增效，全面加快新旧发展动能转换

一是以去产能腾出转型发展新空间。严格执行环保、能耗、质量、安全等标准，依法依规依程序推动钢铁等过剩产能退出市场，确保9月底前压减炼钢产能1006万吨、炼铁产能570万吨、煤炭产能150万吨，10月底前压减焦炭产能360万吨，年底前关停2台、1.2万千瓦煤电机组，5家钢铁企业产能出清。平衡去产能、保就业和防风险的关系，全力做好职工安置和债务处置工作，确保社会可承受、民生有保障。二是以新技术新产品新模式推动传统产业迈向中高端。持续抓好技术改造、新产品开发、钢铁深加工和两化融合等“四个一百”项目，年内完成投资700亿元以上，稳步实施城市建成区和生态红线控制区重点企业搬迁改造，实现钢铁产业优化布局、量减质增。大力发展装配式建筑产业。抓实唐山“钢铁谷”建设，支持京津冀钢铁联盟迁安协同创新研究院加快发展。三是以科技成果“唐山产业化”加快新兴产业集聚。加大科技招商力度，年内实施京津科技成果转化项目100个以上，新增科技型中小企业1000家、科技小巨人企业60家、高新技术企业40家。着力打造先进装备制造、新材料、生物医药等新兴产业集群。抓实“双创”城市示范，年内新增市级以上工程技术研究中心和重点实验室15个、科技企业孵化器(众创空间)10个以上。

3、实施降本增效和创新驱动发展，打造实体经济“升级版”

一是精准打好降本增效“组合拳”。持续开展百家成长性企业“登台阶”工程和扶助中小微企业专项行动，搭建银企对接、产销衔接平台，加快推进个转企、小升规、规改股、股上市，年内新增规模以上工业企业130家以上。落实减税降费政策要求，降低各类交易成本特别是制度性交易成本。实施市县两级领导入企帮扶，建立企业运行监测“一张表”，一企一策破解瓶颈制约，以精准服务提升实体经济发展效益。二是深入做好实体经济与资本融合的乘法。引导更多企业通过挂牌上市降低融资成本、扩大市场销售，力争全年新增“新三板”及以上挂牌上市企业20家以上。支持重点企业开展股权合作。整合地方水泥企业。探索建立“政银保企担”合作模式和“金融超市”服务平台，让更多的金融活水流向实体经济。三是加快实施质量强市品牌战略。围绕先进装备制造、工业消费品和食品等重点领域，深入开展“增品种、提品质、创品牌”专项行动，以产品升级、品牌增值提高企业竞争力。四是持续激发企业家内生动力。继续实施企业家素质提升工程，引导帮助企业家以国际视野、工匠精神做精产品、做强品牌，打造更多百年老店和常青企业。

4、开掘释放比较优势，推进沿海经济扩量提质升级

一是临港产业要聚焦项目落地开工。加快推进精品钢铁、装备制造、现代化工等产业向沿海布局，年内沿海板块实施超亿元项目500个以上，完成投资1000亿元以上。曹妃甸区、海港开发区要在项目推进上体现“沿海速度”。二是重大开放平台要加速产业聚集。举全市之力推动国家级石化产业基地建设。中日韩循环经济示范基地要加快推进。着力释放综合保税区开放功能，加快实施一批国际贸易、出口加工等外向型项目。用足用好天津自贸区可复制政策，持续扩大京冀曹妃甸协同发展示范区产业规模，尽快引进一批北京产业转移项目。健全完善津冀(芦·汉)协同发展示范区与天津对接机制。三是港口发展要突出量效齐升。加快丰南港区起步工程等基础设施建设，开通京唐港区至日本横滨、名古屋等集装箱新航线和外贸内支线，唐山港货物吞吐量达到5.35亿吨，集装箱达到245万标箱、增长26%。优化港口贸易结构，拓展加工、配送、贸易等服务业态。依托唐曹铁路等疏港通道，拉近中心城区与临港区域距离，促进“三大板块”协同互动发展。拓展延伸港口腹地，曹妃甸保税区码头等10个泊位及京唐港区B型保税物流中心建成运营，在新疆、内蒙古、宁夏等地新建3至5个内陆港。

5、释放重大活动综合效应，促进现代服务业提速突破

一是聚焦吸引外地客流和打响特色品牌做精旅游业。以举办全市首届旅发大会和中国(唐山)工业旅游大会为契机，整合拓展开滦国家矿山公园，以新业态盘活弯道山陶瓷创意文化园和启新1889片区，启动中国机车与铁路源头游，改造建设兼具文旅功能的工业博物馆、陶瓷博物馆，争创国家工业旅游城市。开发打造一批体现唐山文化、让游客喜爱的商品，丰富旅游供给。支持迁安、迁西、遵化创建国家全域旅游示范区，推动唐山湾国际旅游岛打造京津冀旅游协作示范区。推动南湖、菩提岛创建国家5A级景区，力争世园会园址、唐山湾国际旅游岛通过国家生态旅游示范区、国家级旅游度假区验收。二是聚焦国际标准和市场化水平做强会展业。坚持政策引导、市场运作，着力培育一批轨道交通、特种机器人、新材料、冶金及装备制造等与我市转型升级相契合的行业展会，吸引具有较强国际影响力的品牌展会永久落户唐山，加快打造国际知名的展会城市。完善提升陶博会、体博会等现有展会，办好第27届全国书博会唐山展会暨第9届河北省书博会、河北教

育装备展等特色展会。年内谋划举办各类展会60次以上，会展业直接收入增长15%以上。三是聚焦开掘优势潜力和市场需求做大文化产业。加快把文化资源转化为文化产业，壮大文化创意设计、文化装备制造等八大产业，推进重点项目建设，培育打造一批文化强县、特色小镇、产业园区和市场主体，促进文化产业与旅游业、互联网等融合发展，年内文化产业增加值增长15%以上。办好中拉沙滩足球锦标赛，吸引社会资本参与场馆建设、足球培训和赛事举办。引进实施一批具有消费引领性的健身休闲项目，举办唐山国际马拉松等高水平赛事活动5项以上，体育产业增加值增长18%以上。四是聚焦提升产业集中度和信息化水平做优商贸物流业。引导传统商业拥抱互联网，提升5大商圈辐射力，打造20个特色电商平台，全年批发、零售业销售额分别增长15%和13%。培育一批行业楼、商务楼、创意楼，年内打造税收超亿元楼宇2栋以上、超5000万元5栋以上。加快路北区友谊路创新创业大街建设。引导优质资源向10大省级物流产业聚集区集中，物流业增加值增长8%以上。坚持客货并举、商旅并重，三女河机场旅客吞吐量突破50万人次、货邮吞吐量达到3000吨。五是聚焦拓展业态和服务实体经济做活金融业。推动唐山银行提质增效，增强竞争实力，支持燕赵财险、唐山农商银行、金发集团、冀唐资产管理公司做大做强。推进县域农村信用社改制农商银行。筹建新型金融业态聚集区，引进融资租赁、第三方财富管理等新兴业态，确保广发银行等金融机构落户。加强金融秩序管理，营造健康活跃金融生态。年内金融业增加值增长15%以上。

6、打造城市热点和北京非首都功能承载地，拓展城市发展新空间

以开放视野、市场思维提升“三城联动”水平，推动城市发展出品位、出高度。围绕缩小政策梯度差拓展产业空间，打造北京非首都功能疏解集中承载地和微中心。围绕提升市民宜居度拓展生活空间，完善城市公共服务功能。围绕增强城市品位内涵拓展公共空间，建设城市标志性片区。围绕盘活资源拓展城市资产增值空间，提高经营城市水平。围绕推进新型城镇化拓展发展空间，实现县域经济优势互补协调并进。

7、深化农业供给侧结构性改革，加快培育农业农村发展新动能

在保证粮食安全前提下，调优产品结构、调好生产方式、调顺产业体系，推动现代农业全环节升级、全链条增值。年内农业产业化经营率达到69%。加快发展农产品精深加工。重点实施遵化中粮肉食加工、玉田同仁堂中药制剂等投资千万元以上农业产业化项目200个，其中超亿元项目10个以上。打造玉田、曹妃甸等5个市级农产品加工园区，新增规模以上农产品加工企业10家以上，全市农产品加工业产值达到660亿元。二是加快打造既“叫好”又“叫座”的品牌农产品。深度挖掘产品价值，狠抓品牌农业创建，不断提升农产品附加值和市场占有率，年内“三品一标”和品牌农产品占比达到50%以上，创建农业品牌20件，申报驰名商标1件、著名商标5件。创建国家农产品质量安全市，12个农业县(市、区)全部纳入国家或省级创建试点。三是加快培育农村新产业新业态。积极开发休闲农业和乡村旅游产品、路线，培育国家级休闲农业星级企业、美丽休闲乡村品牌3至5个，乡村旅游示范点10家。实施电商进村、信息入户项目，加强与“淘宝”等知名电商平台合作，开辟农民增收致富新路径。四是加快创建现代农业“三园一体”。推动现代农业园区、山区综合开发示范沟提档升级，着力打造滦县燕山果品科技产业园区、迁西国家板栗公园等一批现代农业产业园、科技园、创业园和田园综合体，带动农业规模经营、农民创业创新。年内争创省级现代农业园区5个，新增专业大户110个、示范家庭农场40个以上，培育新型经营主体带头人2000人。五是加快美丽乡村建设。持续开展“四美五改·美丽乡村”行动，统筹推进民居改造、垃圾处理等12个专项行动，年内完成304个省级美丽乡村建设，打造10个重点片区。

8、抓好重点领域改革落实落地，增强经济发展内生动力

始终把改革摆在引领全局的突出位置，不断拓展重点领域和关键环节改革的深度广度，加快释放改革红利。深化行政审批制度改革。扎实推进“放管服”，继续取消下放一批审批事项，年内实现市县两级行政审批局全覆盖。深化国有企业改革。推动市属国企脱困发展。抓紧推动开滦等国企“三供一业”分离移交，加快实现市本级国有资产集中监管全覆盖。深化投融资体制改革。发挥政府投资的引导作用和放大效应，在垃圾、污水处理新建项目中强制应用PPP模式。推进市属融资平台公司市场化转型，不断提升运营管理水平。及时修订完善权力清单和责任清单，强化事中事后监管，激发民间投资潜力和创新活力。深化农村综合改革。落实农村承包地“三权分置”，年内完成农村承包地和集体建设用地、宅基地确权颁证工作。完善农村产权交易中心运营机制，扩大农村产权抵押融资试点。加快推进农村股份合作制经济发展，年内新增省级示范组织10个以上。做好国有林场和农垦改革，抓好供销社改革和供销合作集团管理运营，打造社会化便民服务新平台。

9、扩大对外开放合作，提升经济外向度和城市国际化水平

加快培育开放型经济增长点，打造与国际接轨的全方位开放新格局。一是以构建开放型经济新体制激发国际化活力。扎实推进全国构建开放型经济新体制试点建设，坚决破除与国际化沿海强市建设不相适应的体制机制障碍。二是围绕扩大招商引资实效提升国际化水平。瞄准世界500强、国内500强和大企业、大集团，开展小团组招商、以商招商，全年签约亿元以上项目150个以上。深化与京津产业协作，引进实施北京(滦南)国际大健康产业园等重点合作项目200个以上、完成投资300亿元以上。引进省外资金增长10%。三是通过拓展外贸出口市场强化国际化支撑。做大做强卫生陶瓷、先进装备及优质农产品等特色出口基地，争创5个国家级出口质量安全示范区。加大对出口企业融资退税、境外参展的支持力度，鼓励企业多拿出口订单、开拓国际市场，推动外贸出口回稳向好。年内，钢铁、陶瓷、机电、农产品出口额分别增长6%、10%、10%和9%。四是立足深化对外交流合作加速国际化进程。支持钢铁、装备制造等行业企业开展国际合作，加快推进丰南华通线缆喀麦隆生产基地等重点项目，年内新增对外投资企业25家。巩固扩大重大活动期间对外交流成果，加强产业对接合作和民间交往，推动唐山与中东欧、拉美等国家地区城市缔结友好关系。

10、抓好生态环保治理，以更大力度推动环境质量持续好转

实施最严格的环境保护制度，精准推进大气、水和土壤污染防治。一是深入开展“蓝天行动”。持续推进工业污染深度治理，完成焦化行业脱硫脱硝和68家重点企业综合整治，确保8月底前钢铁企业达到特别排放限值要求。强力实施压煤攻坚，全年削减煤炭消费220万吨，全面取缔燃煤小锅炉、茶浴炉和流动燃煤灶具，年内主城区实现散煤清零，路南、路北、高新区完成全域气代煤、电代煤。持续开展“零点行动”“利剑斩污”专项行动，严格治理散料堆场、秸秆垃圾焚烧、露天烧烤、餐饮业油烟排放等点面源污染。规范治理“小散乱污”企业，依法严厉打击偷排偷放行为。年内，PM2.5平均浓度同比下降12.2%以上、达到65微克/立方米左右。二是强力实施“碧水行动”。开展还乡河、沙河等重点河流综合整治，全面清理邱庄水库网箱养鱼，确保国控、省控重点河流湖库断面达到环境功能区划要求。强化饮用水源地保护，完成陡河水库封闭工程，升级改造县级污水处理厂，所有工业园区建成污水集中处理和在线监控设施。狠抓地下水超采综合治理，全面开展地下水污染防治。加强环城水系、城区河道的日常管理，彻底消除城市黑臭水体。三是有序推进“净土行动”。开展土壤环境调查，建立完善土壤环境监测体系。突出抓好城市污染场地、重污染工矿企业、受污染农用地专项整治，市中心区污水处理厂污泥无害化处置率达到100%。加强露天矿山生态修复，开展绿色唐山攻坚，创建国家森林城市。年内新造林34.3万亩以上，森林覆盖率达到37.4%。

11、办好民生实事，确保发展成果更多更公平惠及群众

今年市财政安排资金105.8亿元用于民生和社会事业，占市本级一般预算支出的76.4%，确保让人民群众拥有更多可观可享的获得感。倾力扶持就业创业。持续开展“创业就业三年行动”，突出抓好高校毕业生、农民工和退役军人等重点群体就业，扎实做好去产能职工安置，全年城镇新增就业9.75万人。大力提升社会保障水平。稳步提高城乡居民基础养老金标准，年内启动市中心区5个居家养老服务试点建设。扩大省内定点单位就医直接结算范围。深化城市公立医院综合改革，提升基层医疗服务水平，力争县域内就诊率达到90%。新妇幼医院、市中心医院年内运营开诊。办理居民小区不动产权证3.4万户，新开工保障性住房1.47万套。推广免费婚前医学检查和新生儿产前唐氏筛查，关爱孤儿和病残、流动留守等困境儿童。致力优化教育资源供给。新建、改扩建农村薄弱学校43所，启动智慧校园试点建设。支持华北理工大学、唐山师范学院争创国家一流学科和特色专业，确保西南交大研究生院招生;支持唐山学院以争取更名唐山交通学院为契机，提升办学层次，打造特色鲜明的地方交通大学;支持唐山职业技术学院改善办学条件，提高办学质量;支持唐山工职院申办中华职业大学，曹妃甸国际职教城实现高职院校挂牌招生。支持高中学校适应高考改革及早进行师资储备。着力推动文化和体育事业蓬勃发展。实施文化惠民工程，加强公共文化设施建设，支持民办公益性文化机构发展，探索实行政府购买公共文化服务。推进全民阅读，建设“书香唐山”。做好大剧院、群艺馆、图书馆等运营管理，推进“冀东文艺三枝花”等文化资源走向市场。加强清东陵等文化遗产和历史文物保护。大力开展全民健身活动，新建小型公共运动场5个、健身路径300套。在南湖核心区域新建、改建健身步道12公里，满足群众运动休闲需求。全力保障社会和谐稳定。深化公共安全体系建设，完善“块数据”平台，严厉打击违法犯罪活动。深化重点领域专项整治，坚决遏制重特大安全事故发生。加强食品药品安全监管，争创国家食品安全城市。扎实做好双拥优抚、民族宗教、广播电视、新闻出版、外事侨务、人民防空、老区建设、防震减灾、气象档案等各项工作。

坚持以实干为导向全面加强政府自身建设

强化责任意识，以强烈的使命担当展现加快建设国际化沿海强市的新作为。奋力争取“三个走在前列”、加快实现“三个努力建成”、建设国际化沿海强市，是新一届政府矢志不渝的奋斗目标。全体政府工作人员必须牢记责任使命，撸起袖子加油干，以“赶考”的状态抓发展、促转型、惠民生，尽心尽职尽责地干好每一天、做好每件事，在“爱唐山、做贡献”中创造无愧于岗位、无愧于人民的业绩。

勇于开拓创新，以敢为人先的气魄推动唐山改革发展实现新突破。把思想解放、改革创新作为攻坚克难突破口，努力争做“把信送给加西亚”的人，确保把看似干不成、过去干不成、别人干不成的事情，想方设法干成干好、干出实效。坚持事争一流、高位对标，深入开展机关作风整顿，强化行政管理能力，用政府权力的“减法”换取企业效益的“加法”、市场活力的“乘法”。

弘扬实干作风，以谋实抓深的韧劲精准推动各项工作取得新成效。认真践行“四个干”抓落实机制，对每项工作都具体抓、深入抓、精准抓，特别是对于重大项目建设、化解钢铁产能、大气污染防治等项工作，坚持明责尽责问责，构建上下贯通、层层落实的责任链条。加强督导检查，确保中央和省、市各项决策部署落地生根见效。

坚持依法行政，以权责法定的思维提升政府治理新水平。带头学法尊法守法用法，把法治理念贯穿政府工作全过程。认真办理人大代表建议和政协委员提案，主动接受各界监督，推进重大决策科学化、民主化、法制化。深入开展“七五”普法，全面深化法治唐山建设。大力推进基层行政执法规范化，全力维护社会公平正义，让法治阳光照亮唐山每个公民的生活。

严守纪律规矩，以落实全面从严治党要求打造清正廉洁政府新形象。牢固树立“四个意识”，坚定不移向以习近平同志为核心的党中央看齐。持续抓好“四风”整治，严控“三公”支出，用政府的“紧日子”换取群众和企业的“好日子”。严格落实党风廉政建设责任，强化行政监察和审计监督，严惩不正之风和腐败问题，努力营造清朗清明、干净干事的政治生态。
